# E and prM proteins of genotype V Japanese encephalitis virus are required for its increased virulence in mice

**DOI:** 10.1016/j.heliyon.2019.e02882

**Published:** 2019-11-23

**Authors:** Shigeru Tajima, Ken-ichi Shibasaki, Satoshi Taniguchi, Eri Nakayama, Takahiro Maeki, Chang-Kweng Lim, Masayuki Saijo

**Affiliations:** Department of Virology 1, National Institute of Infectious Diseases, 1-23-1 Toyama, Shinjuku, Tokyo, 162-8640, Japan

**Keywords:** Genetics, Microbiology, Virology, Japanese encephalitis virus, Genotype V, E protein, prM protein, Pathogenicity, Reverse genetics, Infectious cDNA clone

## Abstract

We previously showed that the Japanese encephalitis virus (JEV) genotype V (GV) strain Muar exhibits significantly higher virulence in mice than the genotype I (GI) JEV strain Mie/41/2002. In this study, we attempted to identify the region responsible for the increased virulence of GV JEV using recombinant intertypic and single mutant JEVs. Intertypic viruses containing the GV E region in the Mie/41/2002 backbone showed increased pathogenicity in mice. The amino acid at position 123 in the E protein (E123) of the Mie/41/2002 and GV JEVs was serine and histidine, respectively. A serine-to-histidine substitution at E123 of the Mie/41/2002 increased its virulence. However, histidine-to-serine changes at E123 in the intertypic mutants with the GV E region remained highly virulent. GV Muar prM-bearing mutants were also highly pathogenic in mice. Our results suggest that the E and prM proteins of GV JEV are responsible for the highly virulent characteristics of GV JEV.

## 1 Introduction

Japanese encephalitis (JE) is a severe central nervous system disease caused by infection with the Japanese encephalitis virus (JEV). JE represents a significant public health problem in Asia, with an estimated 68,000 cases of JE per year resulting in 15,000 fatalities, mostly in children (Tsai, 2000) (Erlanger et al., 2009) (Campbell et al., 2011). JEV belongs to the genus *Flavivirus* in the family *Flaviviridae* and is amplified in a bird and pig-mosquito transmission cycle (Pierson and Diamond, 2013). The infected mosquitoes, mainly *Culex* sp., transmit JEV to humans.

JEV has a single-stranded, positive-sense RNA genome. The JEV genome encodes three structural proteins (C, prM, and E) and seven non-structural proteins (NS1, NS2A, NS2B, NS3, NS4A, NS4B, and NS5) from one open reading frame, and it also has non-coding regions (NCRs) at its 5′- and 3′-terminal ends. JEV strains are classified into five genotypes (GI, GII, GIII, GIV, and GV) on the basis of genomic RNA homology (Uchil and Satchidanandam, 2001) (Solomon et al., 2003). The GIII strains were the most widely distributed in JE-endemic areas until the 1990s. However, in most of these regions the prevalent genotype has since transitioned from GIII to GI (Gao et al., 2013) (Schuh et al., 2014). The first GV JEV (Muar strain) was isolated from a patient with encephalitis in Malaysia in 1952; however, no other GV JEV isolates were subsequently found in over 50 years (Solomon et al., 2003). In 2009, GV JEV was identified in a *Culex* mosquito pool in China, and the infectious virus (XZ0934 strain) was isolated (Li et al., 2011). GV JEV was also detected in mosquitoes in Korea in 2010 (Takhampunya et al., 2011). Moreover, all the JEV genomes detected in mosquito pools collected in Korea after 2012 originated from GV JEV (Kim et al., 2015) (Bae et al., 2018). In Korea GI, GIII, and GV JEV have been endemic in recent years, and it is critical to monitor the dynamics of circulating JEV strains in other JE-endemic areas.

All live attenuated and inactivated JE vaccines that are currently available are derived from GIII strains. Previous reports showed that these JE vaccines might have a reduced ability to induce neutralizing antibodies against GV JEV than against other genotypes (Cao et al., 2016; Tajima et al., 2015). Furthermore, another report revealed that IgG antibodies raised against GV JEV XZ0934 had poor neutralizing ability against GIII JEV (de Wispelaere et al., 2015). These findings raise the possibility that GV JEV is distinct from other JEV genotypes in antigenicity, and the current GIII-derived JE vaccines might not provide adequate levels of protection against GV JEV. The low identity in the amino acid sequences between GV and GIII JEV may be involved in the weak efficacy of the GIII-derived vaccines against GV JEV (Tajima et al., 2015), although more detailed studies are needed to evaluate the efficacy of the current JE vaccines against GV JEV.

Understanding the characteristics of GV JEV is essential for determining the response to emerging GV JEVs. However, the characteristics of GV JEV remain to be fully elucidated, as only two GV JEV strains have been isolated to date and only limited studies have been conducted on these strains. We previously showed that GV JEV Muar has unique features in *in vitro* growth and exhibits increased pathogenicity in mice. The growth ability of Muar in cultured mammalian cells was clearly reduced compared to that of GI JEV Mie/41/2002, and conversely, the neuroinvasiveness of Muar was significantly higher than that of Mie/41/2002 (Tajima et al., 2015). A French group also showed that a recombinant GV JEV of the XZ0934 strain exhibited a higher virulence in mice than recombinant GIII JEV (de Wispelaere et al., 2015). Moreover, the same group also demonstrated that the structural protein region (C-prM-E) of XZ0934 plays a critical role in the increased pathogenicity of XZ0934 (de Wispelaere et al., 2015).

In the present study, we attempted to determine the region in GV JEV responsible for its higher pathogenicity in mice. For this purpose, recombinant intertypic chimeric and missense mutant JEVs in the backbone of the GI JEV Mie/41/2002 strain were produced using a reverse genetics system that we previously established, and these were then used for virulence analysis.

## 2 Materials and methods

### 2.1 Ethics statement

Experiments on mice were performed in accordance with the Guidelines for Animal Experiments Performed at the National Institute of Infectious Diseases (NIID), under approval (no.117141, 118011, 118100, 118151, and 119021) from the Animal Welfare and Animal Care Committee of the NIID, Japan. All efforts were made to minimize pain and distress. The mice infected with JEV were observed daily for adverse reactions and signs of disease. For collection of organ samples, mice were euthanized using isoflurane.

### 2.2 Cell culture

All the cell lines used in this study are maintained at our department. African green monkey kidney Vero cells (strain 9013) and human neuroblastoma IMR-32 cells were cultured at 37 °C in 5% CO_2_ in Eagle's Minimal Essential Medium (MEM) (Sigma-Aldrich, St. Louis, MO) supplemented with 10% heat-inactivated fetal bovine serum (FBS) (Sigma-Aldrich) and 100 U/mL penicillin-streptomycin. Mouse neuroblastoma N18 cells were cultured at 37 °C in 5% CO_2_ in Dulbecco's modified Eagle's medium (DMEM) (Sigma-Aldrich) supplemented with 10% FBS and 100 U/mL penicillin-streptomycin (Thermo Fisher Scientific, Waltham, MA).

### 2.3 Viruses

All the viruses used in this study are maintained at our department. GI JEV strain Mie/41/2002 (GenBank accession no. AB241119), which was isolated from pig serum in Japan in 2002 (Nerome et al., 2007; Tajima et al., 2010), and GV JEV strain Muar (GenBank accession no. HM596272), which was isolated from a patient with encephalitis in Malaysia in 1952, were used (Tajima et al., 2015). The working virus stocks were prepared by amplification in Vero cells.

### 2.4 Recombinant viruses

**Fig. 1:**
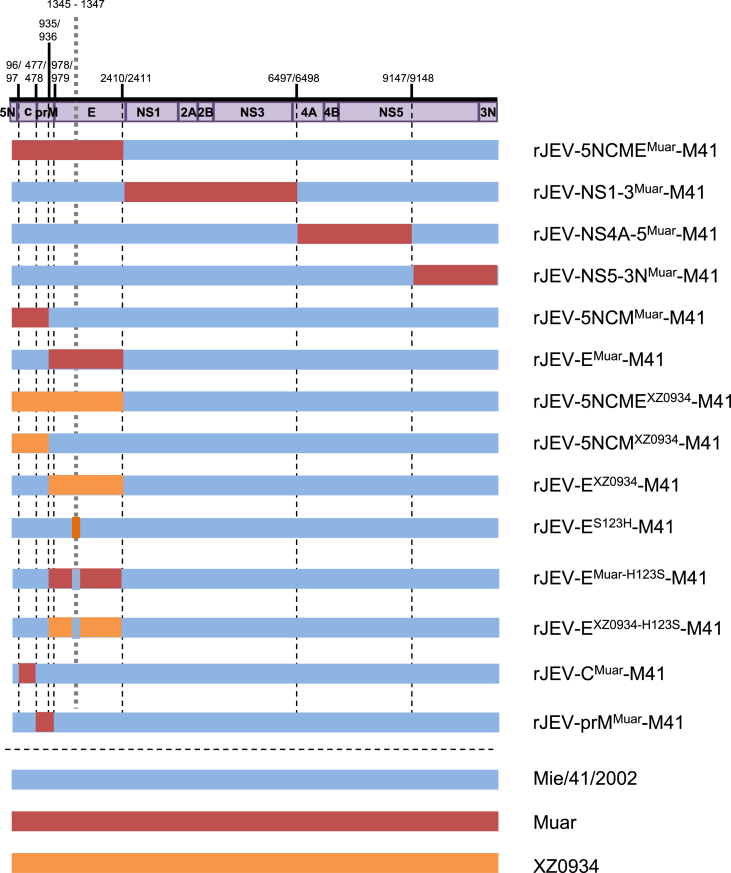
Schematic representation of the genomic structure of the mutant Japanese encephalitis viruses (JEVs) used in this study. The regions derived from GI Mie/41/2002, GV Muar, and GV XZ0934 strains are shown in blue, red, and orange, respectively. Numbers indicate nucleotide positions on the Mie/41/2002 genome. There is no difference in the amino acids at nucleotide positions 936 and 978 among the original three strains.

Fourteen JEV molecular clones of intertypic and missense mutant viruses, namely rJEV-5NCME^Muar^-M41 (5NCME^Muar^), rJEV-NS1-3^Muar^-M41 (NS1-3^Muar^), rJEV- NS4A-5^Muar^-M41 (NS4A-5^Muar^), rJEV-NS5-3N^Muar^-M41 (NS5-3N^Muar^), rJEV-5NCM^Muar^-M41 (5NCM^Muar^), rJEV-E^Muar^-M41 (E^Muar^), rJEV-5NCME^XZ0934^-M41 (5NCME^XZ0934^), rJEV-5NCM^XZ0934^-M41 (5NCM^XZ0934^), rJEV-E^XZ0934^-M41 (E^XZ0934^), rJEV-E^S123H^-M41 (E^S123H^), rJEV- E^Muar−H123S^-M41 (E^Muar−H123S^), rJEV-E^XZ0934-H123S^-M41 (E^XZ0934-H123S^), rJEV-C^Muar^-M41 (C^Muar^), and rJEV-prM^Muar^-M41 (prM^Muar^), were constructed in the Mie/41/2002 backbone, as described previously (Tajima et al., 2010) (Tajima et al., 2015). Briefly, the regions of the infectious cDNA clone rJEV (Mie/41/2002)/pMW119 were replaced with the corresponding regions of the Muar and XZ0934 genomes (Fig. 1). The cDNA fragments of Muar and XZ0934 were amplified by one-step RT-PCR using PrimeScript II High Fidelity RT-PCR kit (Takara Bio, Shiga, Japan) and the primers listed in Supplementary Table S1. A partial cDNA fragment of the XZ0934 strain (5′-NCR-C-prM-E regions, 2412 nucleotides, GenBank accession no. JF915894) was synthesized by Integrated DNA Technologies (Skokie, IL). The amplified fragments were inserted into the corresponding regions by conventional molecular cloning methods using the In-Fusion Cloning system (Takara Bio). The single missense mutations, E^S123H^, E^Muar−H123S^, and E^XZ0934-H123S^, were introduced into the rJEV (Mie/41/2002)/pMW119, rJEV-E^Muar^-M41/pMW119, and rJEV-E^XZ0934^-M41/pMW119 strains, respectively, by inverse PCR-based site-directed mutagenesis (Tajima et al., 2006). The nucleotide sequence of the viral genome region of the recombinant clones were examined after amplification of the plasmids in *E. coli* STBL2 (Thermo Fisher Scientific). The recombinant viruses were recovered by transfecting the Vero cells with *in vitro*-transcribed recombinant viral RNA, as previously described (Tajima et al., 2006). The nucleotide sequence of the recombinant virus was also determined; no nucleotide mutation was detected.

### 2.5 Plaque formation assay and analysis of the growth kinetics

For the plaque formation assay, Vero cells (3 × 10^5^ cells/well) were plated in 12-well plates and inoculated with the viruses for 1h at 35–37 °C. A MEM-based overlay medium containing 1% methyl cellulose and 2% fetal bovine serum was added to the wells and the cells and then incubated for 5 days at 35–37 °C. The cells were fixed using a 10% formalin-PBS solution and subsequently stained with methylene blue as described previously (Tajima et al., 2006).

The growth of the JEV strains was analyzed as described previously (Tajima et al., 2010). Briefly, cells (N18 and IMR-32) were plated in 6-well culture plates and infected with the JEV in MEM supplemented with 2% FBS at a multiplicity of infection of 0.1 plaque forming units (PFU)/cell. Small aliquots of the media were removed at one-day intervals, and their infectious viral titers were determined by a plaque formation assay on Vero cells as described above.

### 2.6 Mouse challenge and sample collection

All animal experiments were conducted in accordance with the Fundamental Rules for Animal Experiments of our institute. Female ddY mice (3-weeks old) were purchased from Japan SLC, Inc. (Shizuoka, Japan) and maintained in a specific-pathogen-free environment.

For virulence analysis, groups of mice (n = 8 or 10) were inoculated intraperitoneally with 1 × 10^3^, 1 × 10^4^, or 1 × 10^5^ PFU (100 μL) of JEV virus solution diluted with a 0.9% NaCl solution. The mice were observed, and the body weight of mice was measured every day for 18 days after inoculation to determine the survival rates. The survival curves were compared using BellCurve for Excel (Social Survey Research Information, Tokyo, Japan) employing the log-rank (Mantel–Cox) test. A *P*-value < 0.01 was considered significant.

For sample collection, groups of mice (n = 5 or 6) were inoculated intraperitoneally with 1 × 10^4^ PFU (100 μL) of JEV solutions. Serum, brain, and spleen were collected from the mice, and the titer and RNA levels of the infectious virus in the samples were measured as described below. Tissue weights were determined, and the tissues were homogenized in 500 μL of MEM with 2% FBS and the homogenate was used for further analyses.

### 2.7 Measurement of viral titer

Viral titers for each sample were determined by plaque forming assay as described above and then statistically compared by using BellCurve for Excel, employing the Mann–Whitney U test. A *P*-value < 0.01 was considered significant.

### 2.8 Measurement of viral-genome copy number by using real-time RT-PCR

Total RNA was extracted from each tissue sample by using a High Pure Viral RNA Purification Kit (Roche Diagnostics, Mannheim, Germany). JEV RNA was quantified by quantitative real-time RT-PCR with Fast Virus One-Step Master Mix (Thermo Fisher Scientific). The JEV genome was amplified using primers JE-Multi-f (5′-AGAACGGAAGAYAACCATGACTAAA-3′) and JE-Multi-r (5′-CCGCGTTTCAGCATATTGAT-3′), and probe JE-Multi (5′-FAM-ACCAGGAGGGCCCGG-MGB-3′) (Shirato et al., 2005). Genome copy numbers were statistically compared using BellCurve for Excel, employing the Mann–Whitney U test.

## 3 Results

### 3.1 Pathogenicity of the GI-GV intertypic mutants, 5NCME^Muar^, NS1-3^Muar^, NS4A-5^Muar^, and NS5-3N^Muar^, in mice

We previously showed that the GV strain Muar is significantly more pathogenic than the GI strain Mie/41/2002 (Tajima et al., 2015). To specify the genomic region on GV involved in its high virulence phenotype, four intertypic mutant viruses incorporating the Mie/41/2002 backbone, 5NCME^Muar^, NS1-3^Muar^, NS4A-5^Muar^, and NS5-3N^Muar^, were produced (Fig. 1). Mice were intraperitoneally inoculated with Mie/41/2002, Muar, and the four mutant viruses (1 × 10^4^ PFU/mouse) and observed for 18 days (Table 1; Supplementary Fig. S1, Experiment 1). None of the mice inoculated with Mie/41/2002 died, but all the eight mice inoculated with Muar died. All the eight mice inoculated with 5NCME^Muar^ died, and the mean time to death of 5NCME^Muar^-inoculated mice was equivalent to that of Muar-inoculated mice. In contrast, in the case of both the NS1-3^Muar^- and NS5-3N^Muar^-inoculated mice, two out of eight mice died. Although a loss in body weight was observed in some NS4A-5^Muar^-inoculated mice (Supplementary Fig. S2), these mice did not die. These results indicate that the 5′-NCR-C-prM-E region of Muar is mainly responsible for its high virulence.

**Table 1:** Mouse neuroinvasiveness of recombinant JEV strains.

Experiment no.	Virus	Dose (pfu/mouse)	Survival^a^	Mean time to death (day)	*P* value^b^ (vs Mie/41/2002)	*P* value^b^ (vs Muar)	*P* value^b^ (vs rJEV-E^Muar^-M41)	*P* value^b^ (vs rJEV-E^XZ0934^-M41)	*P* value^b^ (vs rJEV-5NCM^Muar^-M41)
1	Mie/41/2002	10^4^	8/8	-	-	<0.001*			
	Muar	10^4^	0/8	7.8	<0.001*	-			
	rJEV-5NCME^Muar^-M41	10^4^	0/8	7.8	<0.001*	1.0			
	rJEV-NS1-3^Muar^-M41	10^4^	6/8	16.0	0.1435	<0.001*			
	rJEV-NS4A-5^Muar^-M41	10^4^	8/8	-	1.0	<0.001*			
	rJEV-NS5-3N^Muar^-M41	10^4^	6/8	15.6	0.1435	<0.001*			

2	Mie/41/2002	10^4^	8/8	-	-				
	rJEV-5NCME^Muar^-M41	10^4^	0/8	6.4	<0.001*				
	rJEV-5NCM^Muar^-M41	10^4^	1/8	8.5	<0.001*				
	rJEV-E^Muar^-M41	10^4^	1/8	8.6	<0.001*				
	rJEV-5NCME^XZ0934^-M41	10^4^	0/8	6.3	<0.001*				
	rJEV-5NCM^XZ0934^-M41	10^4^	2/8	10.9	0.002*				
	rJEV-E^XZ0934^-M41	10^4^	1/8	10.1	<0.001*				
	rJEV-E^S123H^-M41	10^4^	3/8	11.3	0.009*				

3	Mie/41/2002	10^3^	9/10	17.0	-				
	rJEV-E^S123H^-M41	10^3^	10/10	-	0.3173				
	Mie/41/2002	10^5^	5/10	12.6	-				
	rJEV-E^S123H^-M41	10^5^	0/10	6.8	0.007*				

4	rJEV-E^Muar^-M41	10^4^	3/10	10.6			-		
	rJEV-E^Muar-H123S^-M41	10^4^	1/10	9.0			0.4773		
	rJEV-E^XZ0934^^-^M41	10^4^	3/10	10.5				-	
	rJEV-E^XZ0934^^-H123S^-M41	10^4^	4/10	13.0				0.38	

5	rJEV-5NCM^Muar^-M41	10^4^	2/8	11.0					-
	rJEV-C^Muar^-M41	10^4^	7/8	16.8					0.021
	rJEV-prM^Muar^-M41	10^4^	2/8	11.8					0.9093

a No. of mice surviving/no. of mice inoculated.

b *P* value relative to Mie/41/2002, Muar, rJEV-E^Muar^-M41, rJEV-E^XZ0934^-M41, and rJEV-5NCM^Muar^-M41 by log-rank test. Asterisks indicate statistical significance (*, *p* < 0.01).

### *3.2* *In vitro* growth characteristics of the 5NCME^Muar^ mutant

**Fig. 2:**
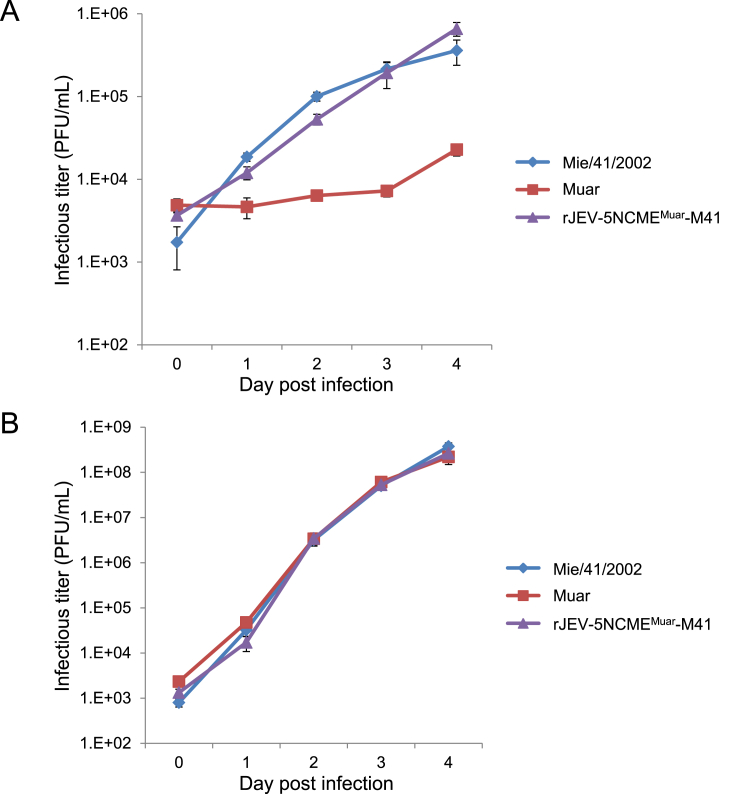
Growth properties of GI Mie/41/2002, GV Muar, and mutant rJEV-5NCME^Muar^-M41 in mouse neuroblastoma N18 (A) and human neuroblastoma IMR-32 (B) cells. Values represent the mean and standard deviation for three independent tests.

Next, the *in vitro* growth properties of the 5NCME^Muar^ mutant were examined. Previously, we have shown that the growth ability of Muar in mouse neuroblastoma N18 cells is significantly lower than that of Mie/41/2002 (Tajima et al., 2015). Although the growth kinetics of 5NCME^Muar^ in N18 cells were very similar to those of Mie/41/2002, these were clearly higher than those observed for Muar (Fig. 2A). However, in human neuroblastoma IMR-32 cells, no significant difference was observed in the proliferation rates among the three strains (Fig. 2B). These results suggest that the replacement of 5′-NCR-C-prM-E region of Mie/41/2002 by that of Muar does not affect the *in vitro* growth characteristics of Mie/41/2002.

### 3.3 Pathogenicity of the 5NCM^Muar^, E^Muar^, 5NCME^XZ0934^, 5NCM^XZ0934^, and E^XZ0934^ mutants in mice

To identify the genes involved in the high virulence of Muar, additional intertypic viruses, 5NCM^Muar^ and E^Muar^, which contain the 5′-NCR-C-prM region and only the E region of Muar, respectively, were produced and inoculated into mice (1 × 10^4^ PFU/mouse) (Fig. 1; Table 1; Supplementary Fig. S1, Experiment 2). Seven out of eight mice inoculated with 5NCM^Muar^ and E^Muar^ died, and the mean times to death of the two groups were very similar, indicating that both the 5′-NCR-C-prM and E regions are involved in the virulence of Muar.

Three novel mutant viruses, 5NCME^XZ0934^, 5NCM^XZ0934^, and E^XZ0934^, which contain the 5′-NCR-C-prM-E region, 5′-NCR-C-prM region, and only the E region of the recent GV isolate XZ0934, respectively, were produced and then inoculated into mice (Fig. 1; Table 1; Supplementary Fig. S1, Experiment 2). All the eight mice inoculated with 5NCME^XZ0934^ or 5NCME^Muar^ died, and the mean time to death of 5NCME^XZ0934^-inoculated mice was very similar to that of the 5NCME^Muar^-inoculated mice. Six and seven out of eight mice inoculated with 5NCM^XZ0934^ and E^XZ0934^, respectively, died, and the mean times of death in the two mice groups were similar. These results imply that the 5′-NCR-C-prM-E region in XZ0934 also has the ability to increase the virulence of Mie/41/2002.

### 3.4 Growth of the Mie/41/2002, Muar, 5NCM^Muar^, and E^Muar^ strains in mice

**Fig. 3:**
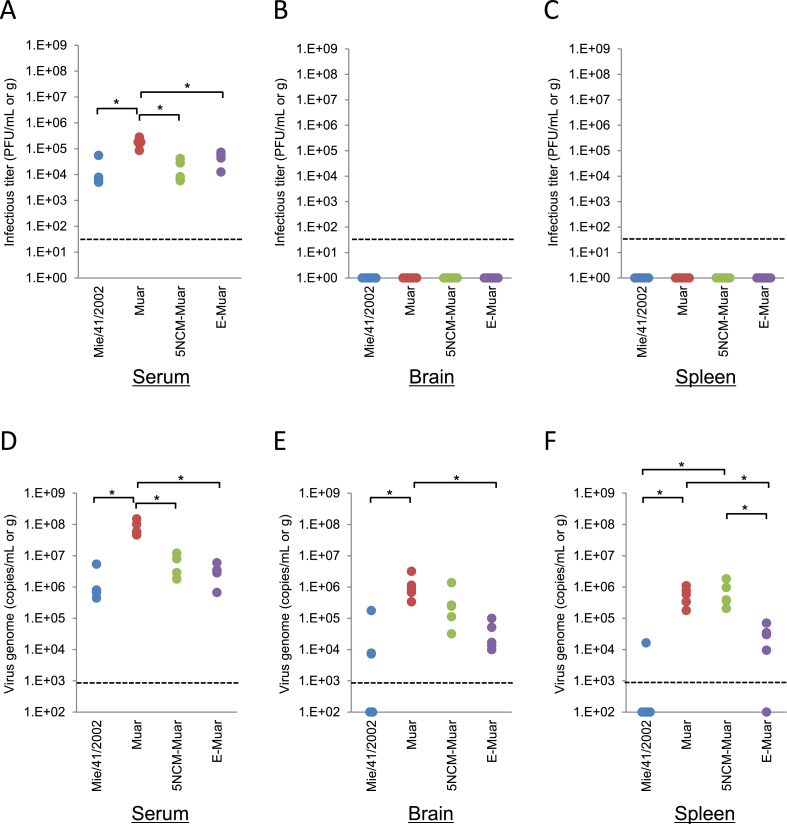
Levels of infectious virus and viral RNA at two days after inoculation in JEV-infected mice. Mice inoculated with Mie/41/2002 (n = 5), Muar (n = 5), rJEV-5NCM^Muar^-M41 (5NCM-Muar) (n = 5), or rJEV-E^Muar^-M41 (E-Muar) (n = 5) were euthanized at two days after inoculation, and serum, brain, and spleen samples were collected. Sera and tissue homogenates were used to quantify the titer of the infectious virus (PFU/mL or g) (A–C) and the viral genome (genome copies/mL or g) (D–F). Dotted line: detection limit; solid lines: mean values. Significance was analyzed using the Mann–Whitney U test (*p < 0.01).

Viremia and infectious virus levels in Mie/41/2002-, Muar-, 5NCM^Muar^- and E^Muar^-inoculated mice were examined. At two days after inoculation, no infectious virus was detected in the brain and spleen in all the four groups, whereas viremia was seen in all the groups (Fig. 3A-C). The viremia in the Muar-inoculated mice was higher but not as much as compared to the other strain-inoculated mice. Viral RNAs were detected in most of the samples; however, the levels were higher in Muar- and 5NCM^Muar^-inoculated mice (Fig. 3D-F).

**Fig. 4:**
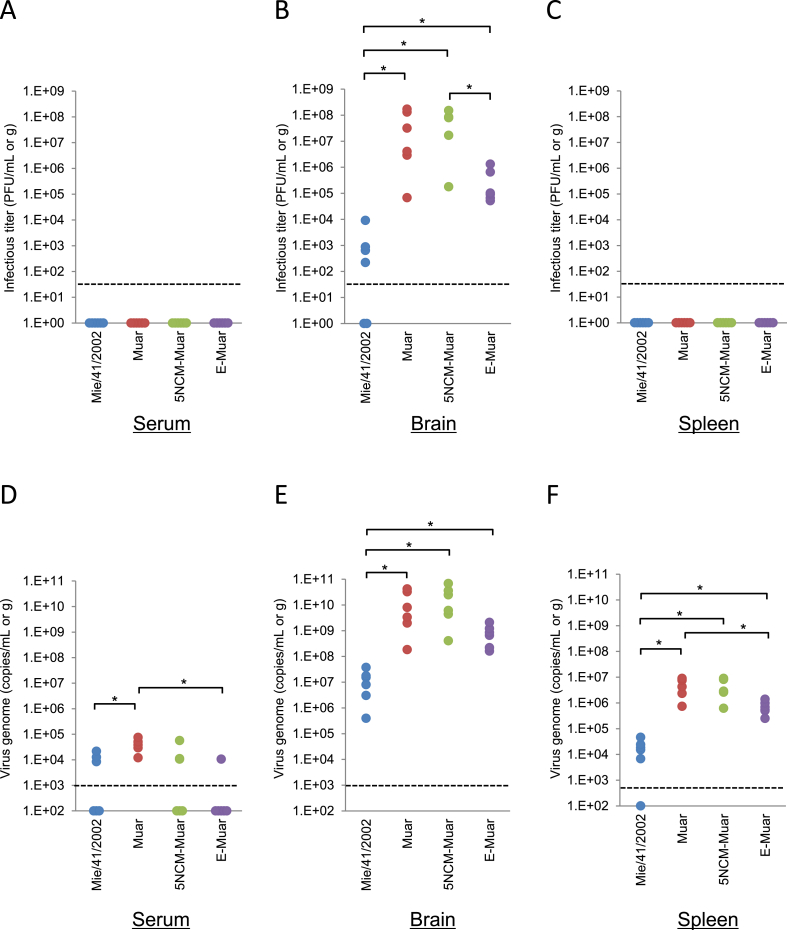
Levels of infectious virus and viral RNA at five days after inoculation in JEV-infected mice. Mice inoculated with Mie/41/2002 (n = 6), Muar (n = 6), rJEV-5NCM^Muar^-M41 (5NCM-Muar) (n = 6), or rJEV-E^Muar^-M41 (E-Muar) (n = 6) were euthanized at five days after inoculation, and serum, brain, and spleen samples were collected. Sera and tissue homogenates were tested for quantifying the infectious virus titer (PFU/mL or g) (A–C) and the viral genome (genome copies/mL or g) (D–F). Dotted line: detection limit; solid lines: mean values. Significance was analyzed using the Mann–Whitney U test (*p < 0.01).

At five days post infection, no infectious virus was detected in any of the serum and spleen samples (Fig. 4A and C). In contrast, higher titers of infectious virus were detected in the brains of mice inoculated with Muar, 5NCM^Muar^, and E^Muar^ compared with Mie/41/2002 (Fig. 4B). In particular, the titers were significantly higher for Muar- and 5NCM^Muar^-inoculated mice. Viral RNA was detected in most of the brain and spleen samples from the mice inoculated with the JEVs, and the levels of viral RNA were higher in the Muar and mutant virus-inoculated mice than in the Mie/41/2002-inoculated mice (Fig. 4D-F).

### 3.5 Effect of single amino acid substitutions at position 123 of the JEV E protein on the virulence in mice

**Fig. 5:**
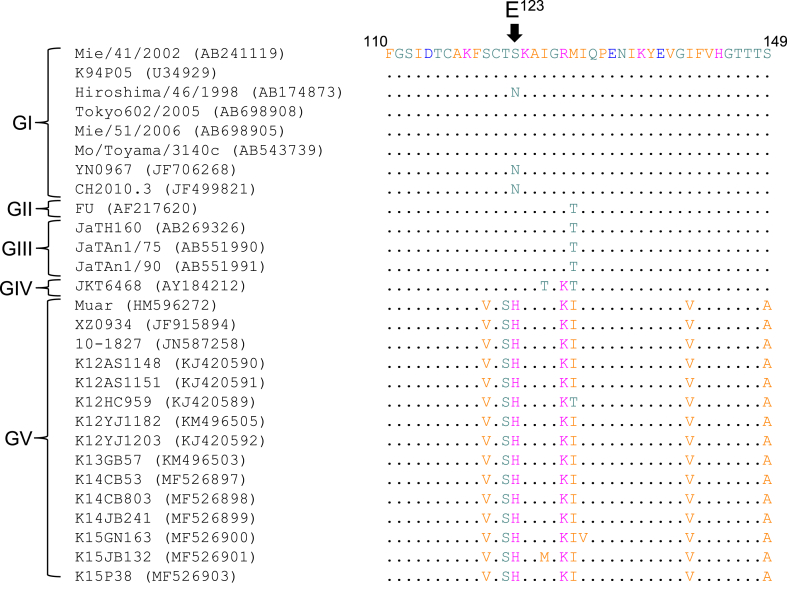
Alignment of partial amino acid sequences of Japanese encephalitis virus (JEV) strains. A partial sequence of the E region between aa 110 and aa 149 is shown. The amino acid sequence of GI Mie/41/2002 is used as a reference sequence. The arrowhead indicates the residue at position 123 of the E protein.

We previously reported that the amino acid at position 123 of JEV E (E123) is involved in the pathogenicity of JEV in mice (Tajima et al., 2010). An amino acid substitution of E123 from serine to arginine increases the virulence of Mie/41/2002, whereas a serine-to-asparagine replacement at that site does not alter the virulence (Tajima et al., 2010) (Yamaguchi et al., 2013). In most of GI, GII, GIII, and GIV JEV strains, the E123 was serine or asparagine (Fig. 5), whereas a histidine residue at E123 was conserved in the GV JEV strains. To assess whether the histidine residue at E123 in the Muar and XZ0934 strains is associated with their virulence, a mutant virus E^S123H^ was prepared in which only the serine at E123 in the Mie/41/2002 was substituted with histidine, and the virus was then inoculated into mice (1 × 10^4^ PFU/mouse) (Table 1; Supplementary Fig. S1, Experiment 2). Five out of the eight inoculated mice died, and the mortality rate of the mice group was significantly higher than that of the Mie/41/2002-inoculated mice. The amount of inoculated virus was also changed to examine the virulence of the E^S123H^ mutant in mice (Table 1; Supplementary Fig. S1, Experiment 3). None of the mice died at low concentrations (1 × 10^3^ PFU/mouse) of the mutant, but all 10 mice inoculated at high concentrations (1 × 10^5^ PFU/mouse) died, and the number of dead mice in the group was significantly higher than in the Mie/41/2002-inoculated mice group. Additionally, the time to death of the E^S123H^-inoculated mice was about half as long as that of the Mie/41/2002-inoculated mice. These results suggest that the histidine residue at E123 on GV JEV E protein is associated with the higher virulence of GV JEV.

To investigate the possibility of involvement in higher virulence of amino acid locations in the GV JEV E protein other than E123, two additional mutant viruses, E^Muar−H123S^ and E^XZ0934-H123S^, were produced and inoculated into mice (1 × 10^4^ PFU/mouse) (Table 1; Supplementary Fig. S1, Experiment 4). No significant differences in the survival rate and time to death were observed between the E^Muar^-infected and E^Muar−H123S^-infected mice, or between the E^XZ0934^-infected and E^XZ0934-H123S^-infected mice. These results suggest that not only E123, but other sites in the GV JEV E protein, contribute to the higher pathogenicity of GV JEV in mice.

### 3.6 Pathogenicity of the C^Muar^ and prM^Muar^ mutants in mice

To clarify whether the C or prM protein of Muar is responsible for the high pathogenicity of this strain, the two mutant viruses, C^Muar^ and prM^Muar^, were produced and the virulence of these mutants in mice was examined (1 × 10^4^ PFU/mouse) (Table 1; Supplementary Fig. S1, Experiment 5). Six out of eight mice inoculated with 5NCM^Muar^ and prM^Muar^ died, and the mortality rates of the two groups were higher than that of C^Muar^-inoculated mice although no clear statistical significance was observed in the survival rates between C^Muar^- and 5NCM^Muar^-inoculated mice groups (*p* = 0.021). This indicates that the prM protein of Muar is also associated with the higher virulence of this strain.

## 4 Discussion

We have previously shown that the GV strain Muar is more pathogenic in mice than the GI strain Mie/41/2002. In this study, we attempted to determine which region is involved in the difference in pathogenicity in mice between the strains. It has been known that structural proteins, especially the E protein, are mainly involved in determining the virulence of JEV (Ni and Barrett, 1998) (Wu and Lee, 2001) (Monath et al., 2002) (Wu et al., 2003) (Chiou et al., 2005) (Mori et al., 2005) (Chambers et al., 2007) (Kim et al., 2008) (Zhou et al., 2018). We also demonstrated that the E protein is involved in the high virulence of the GIII strain Beijing-1 (Tajima et al., 2010). In addition, mutations of amino acids in E protein have been found in live-attenuated JE vaccines and attenuated JEVs (Sumiyoshi et al., 1995) (Ni and Barrett, 1996) (Zhao et al., 2005) (Liang et al., 2009) (Yu, 2010) (Zheng et al., 2018). These findings suggest that the E region may also be related to the high virulence of GV JEV. A French group showed that the GV XZ0934 strain is more pathogenic in mice than the GIII RP-9 strain and that the structural protein region of XZ0934 is involved in its higher pathogenicity (de Wispelaere et al., 2015). However, in this study we did not investigate which of the three structural proteins was associated with the high virulence. Herein, analyses of the intertypic viruses proved that the E and prM regions are involved in the higher pathogenicity of GV JEV.

**Fig. 6:**
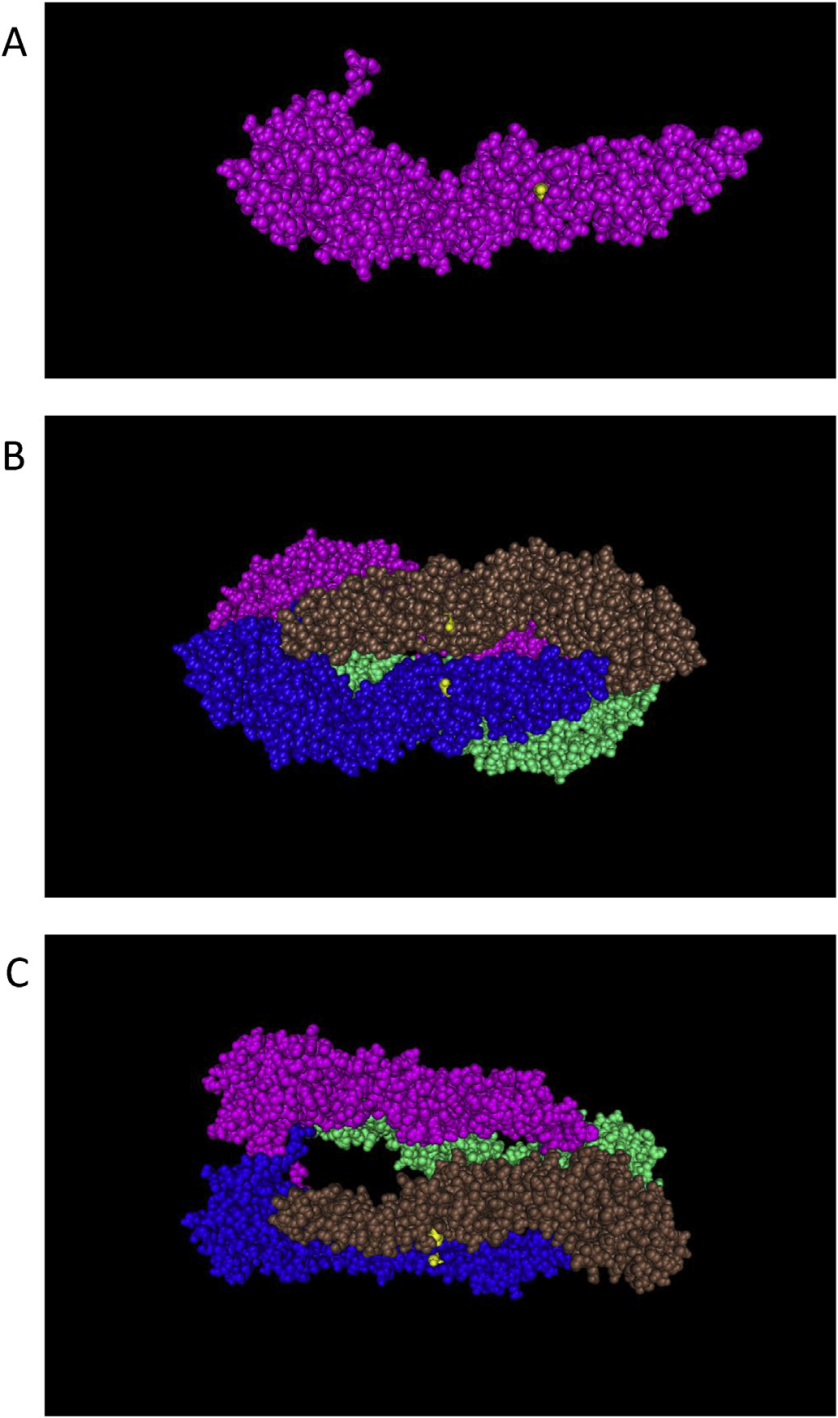
Molecular graphics of the Japanese encephalitis virus (JEV) E protein (SA-14-14-2 strain, PDB accession code 3P54). A: asymmetric unit (monomer). B and C: biological unit (tetramer). Yellow balls: amino acid residue (serine) at position 123 of E protein.

In the E protein, there are about 8% differences in amino acids (about 40 residues) between Mie/41/2002 and GV JEVs. Previous studies on the identification of the sites involved in the attenuation of GIII JEV strains suggested that amino acid changes at positions 107, 138, 176, 177, 244, 264, 279, 315, 366, and 439 of E may be involved (Zhao et al., 2005) (Gromowski et al., 2015; Liang et al., 2009) (Yang et al., 2017) (Zhou et al., 2018) (Zheng et al., 2018). Nine out of 10 amino acid residues at these positions are identical among the three strains used in this study. Amino acid residue at position 366 is different in Mie/41/2002 (serine) and GV JEVs (alanine); however, the residue in GV JEVs is the same as that in the attenuated strain SA-14-14-2 (Gromowski et al., 2015). There are more than 20 amino acid residues unique to GV JEVs in the E region, including E123. The E123 amino acid is serine or asparagine in GI, GII, GIII, and GIV JEV, but histidine in GV JEV (Fig. 5). In this study, we show that a serine-to-histidine substitution at E123 (E^S123H^) increased the virulence of Mie/41/2002. The E protein forms head-to-tail homodimers and consists of three domains: domain I, II, and III. E123 is located in domain II, which is important for homodimerization of the E protein, and amino acid mutations in domain II may influence the virulence by affecting low pH conformational transitions (Rey et al., 1995) (Kolaskar and Kulkarni-Kale, 1999) (Nybakken et al., 2006). It has been suggested that protonation of histidine residues in the E protein at acidic pH affects the structural transition and membrane fusion in the lifecycle of flavivirus (Fritz et al., 2008) (Nelson et al., 2009). In contrast to serine, which belongs to the polar, uncharged side chain group, histidine is classified in the positively-charged side chain group, which also includes arginine. We previously showed that a serine-to-arginine substitution at E123 (E^S123R^) increases the virulence of JEV (Tajima et al., 2010), suggesting that a substitution of serine at E123 to a positively-charged amino acid may contribute to increased virulence of JEV in mice. Crystal structure analysis of the JEV E protein shows that E123 in the domain II is located on the surface of the JEV virion (Fig. 6) (Luca et al., 2012). It is possible that the domain II interacts with cellular factors and amino acid residue of E123 influences the affinity of factors for the domain II. Our report also indicated that the E^S123R^ mutation increases the proliferative capacity in mouse neuroblastoma N18 cells (Tajima et al., 2010). However, the proliferation ability of the Muar strain in N18 cells is lower than that of Mie/41/2002, whereas the growth kinetics of the 5NCME^Muar^ mutant were similar to those of Mie/41/2002. These results suggest that the proliferative potential of JEV in N18 cells is not necessarily reflective of its virulence in mice.

The virulence of the E^S123H^ mutant was slightly lower than that of Muar and intertypic 5NCME and E mutants. Moreover, the virulence of E^Muar−H123S^ and E^XZ0934-H123S^ mutants was similar to that of E^Muar^ and E^XZ0934^ mutants, respectively. These data imply that the histidine residue at E123 is not sufficient to explain the high virulence of GV JEV. Thus, not only the amino acid residue at E123, but other residues unique to GV JEV (for example, E120, E122, E128, E141, and E149 in Fig. 5) may also be involved in the high virulence of GV JEV, and the residues may cooperate with each other to promote the high pathogenicity of GV JEV.

In the present study, we demonstrate that in addition to the E region, the prM region also possesses a site responsible for the high virulence of GV JEV. There are about 10% differences in prM amino acid residues between the Mie/41/2002 and GV strains. Little is known about how the prM protein contributes to the virulence of JEV. However, recent reports suggest that prM of *Zika virus* is involved in its pathogenicity in mice (Yuan et al., 2017) (Kato et al., 2017). The prM of JEV contains an N-linked glycosylation site that is crucial for the pathogenesis of JEV, although the glycosylation motif is conserved among JEV genotypes (Kim et al., 2008). The prM protein interacts with the E proteins to form prM–E heterodimers, which are required for the folding of the E protein and the formation of immature virions (Konishi and Mason, 1993) (Lorenz et al., 2002), suggesting that the replacement of the prM region might influence the assembly and maturation processes of virus particles through the E protein. Identification of the sites responsible for increased pathogenicity of the prM mutant would provide new insights into the biology and pathogenesis of JEV.

In this study, we did not produce an intertypic mutant that has only the 5′-NCR region of GV JEV. There are three and four nucleotide differences in the 5′-NCR between Mie/41/2002 and Muar, and between Mie/41/2002 and XZ0934, respectively (Supplementary Fig. S3). A previous report indicated that a single nucleotide substitution in the 5′-NCR of JEV influences the virulence of JEV in mice (Gromowski et al., 2015). The nucleotide positions in the 5′-NCR of GV JEV might also be associated with the pathogenicity of GV JEV.

The number of JE patients has rapidly increased in Korea since 2010. GV JEV was first identified in Korea in 2010, and most of the JEV identified thereafter are GV strains (Kim et al., 2015). It is unclear whether this rise in the number of JE patients is related to the spread of GV. Invasion of GV has not been confirmed in Japan and other JE-endemic areas besides Korea. In most areas where JE epidemics occur, GI JEVs are persistent, as they were in Korea before 2010; however, GV invasion may cause a rapid shift to GV in these areas, which can increase the number of patients with JE. To prepare for a global epidemic of GV, it is important to further analyze and improve our understanding of GV JEV.

## Declarations

### Author contribution statement

Shigeru Tajima: Conceived and designed the experiments; Performed the experiments; Analyzed and interpreted the data; Contributed reagents, materials, analysis tools or data; Wrote the paper.

Ken-ichi Shibasaki, Satoshi Taniguchi, Eri Nakayama, Takahiro Maeki: Performed the experiments.

Chang-Kweng Lim, Masayuki Saijo: Analyzed and interpreted the data.

### Funding statement

This work was supported by the Research Program on Emerging and Re-emerging Infectious Diseases of the Japan Agency for Medical Research and Development (AMED) under Grant Number JP19fk0108035.

### Competing interest statement

The authors declare no conflict of interest.

### Additional information

No additional information is available for this paper.

## Appendix A Supplementary data

Suppl. Figs. 191007.pptx Suppl. Figs. 191007.pptx

primer list_190703 primer list_190703
