# Supplementary material for: E and prM proteins of genotype V Japanese encephalitis virus are required for its increased virulence in mice
Source: Heliyon. 2019 Nov 23;5(11):e02882. doi: 10.1016/j.heliyon.2019.e02882 (PMC6881638; doi:10.1016/j.heliyon.2019.e02882)
Supplement: Suppl. Figs. 191007.pptx [file mmc1.pptx]

## Slide 1
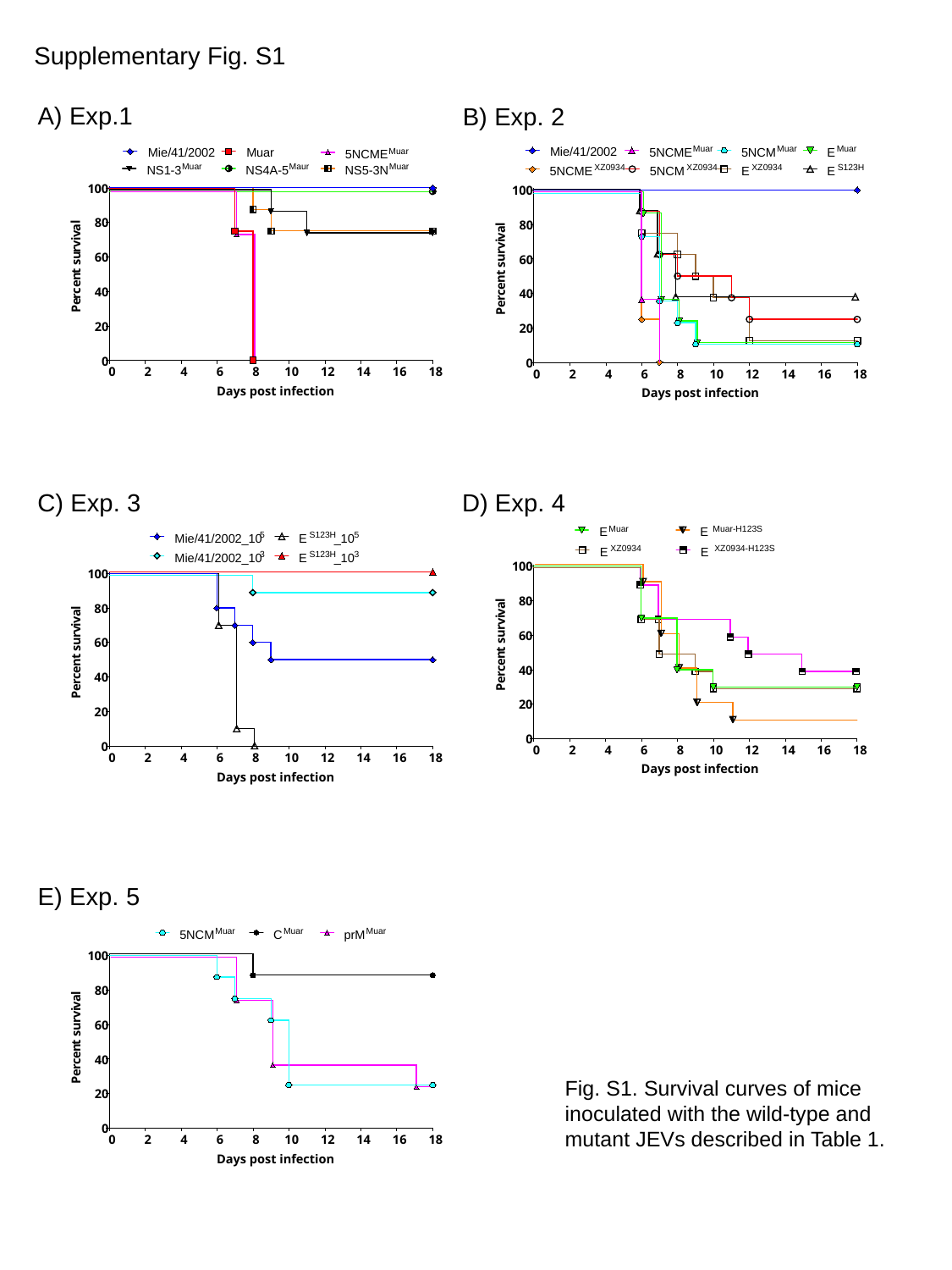

Supplementary Fig. S1
A) Exp.1
B) Exp. 2
Muar
Muar
Muar
Mie/41/2002
5NCME
5NCM
E
XZ0934
XZ0934
XZ0934
S123H
5NCME
5NCM
E
E
100
l
80
a
v
i
v
r
u
60
s
t
n
e
c
40
r
e
P
20
0
0
2
4
6
8
10
12
14
16
18
Days post infection
Mie/41/2002
Muar
Muar
5NCME
Muar
Maur
Muar
NS1-3
NS4A-5
NS5-3N
100
l
80
a
v
i
v
r
u
60
s
t
n
e
c
40
r
e
P
20
0
0
2
4
6
8
10
12
14
16
18
Days post infection
D) Exp. 4
C) Exp. 3
Muar
Muar-H123S
E
E
XZ0934
XZ0934-H123S
E
E
100
l
80
a
v
i
v
r
u
60
s
t
n
e
c
40
r
e
P
20
0
0
2
4
6
8
10
12
14
16
18
Days post infection
5
S123H
5
Mie/41/2002_10
E
_10
3
S123H
3
Mie/41/2002_10
E
_10
100
l
80
a
v
i
v
r
u
60
s
t
n
e
c
40
r
e
P
20
0
0
2
4
6
8
10
12
14
16
18
Days post infection
E) Exp. 5
Muar
Muar
Muar
5NCM
C
prM
100
80
l
a
v
i
v
r
60
u
s
t
n
e
40
c
r
e
P
20
0
0
2
4
6
8
10
12
14
16
18
Days post infection
Fig. S1. Survival curves of mice inoculated with the wild-type and mutant JEVs described in Table 1.

## Slide 2
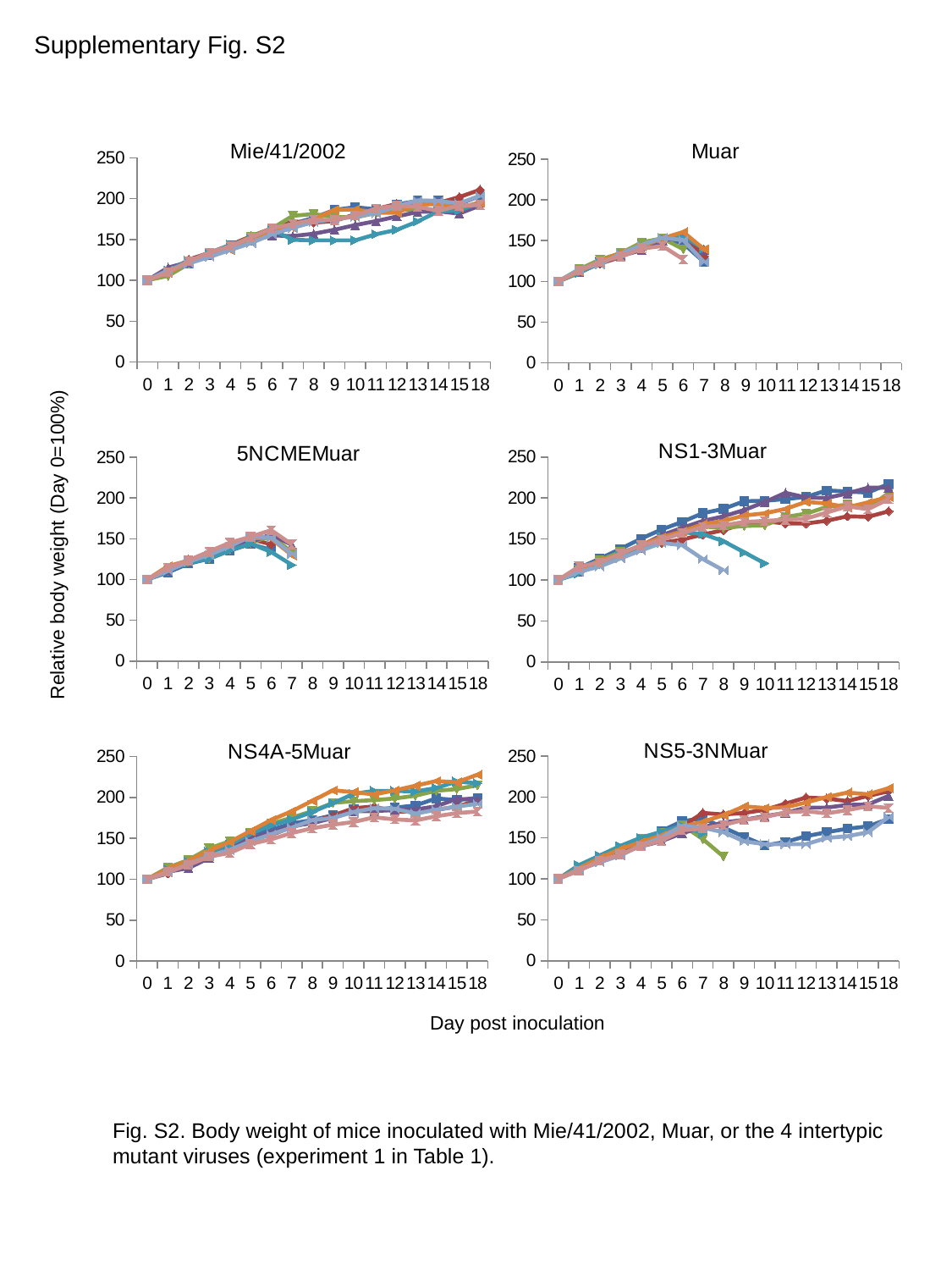

Supplementary Fig. S2
### Chart: Mie/41/2002
| Category | 1 | 2 | 3 | 4 | 5 | 6 | 7 | 8 |
|---|---|---|---|---|---|---|---|---|
| 0 | 100.0 | 100.0 | 100.0 | 100.0 | 100.0 | 100.0 | 100.0 | 100.0 |
| 1 | 111.11111111111111 | 109.55414012738854 | 105.40540540540539 | 115.49295774647888 | 112.24489795918369 | 108.9655172413793 | 112.14285714285714 | 109.92907801418438 |
| 2 | 120.91503267973856 | 125.47770700636943 | 120.27027027027026 | 122.53521126760563 | 123.80952380952381 | 122.06896551724138 | 120.71428571428571 | 123.40425531914893 |
| 3 | 132.6797385620915 | 133.7579617834395 | 131.75675675675674 | 130.98591549295776 | 133.33333333333334 | 131.0344827586207 | 128.57142857142858 | 134.04255319148933 |
| 4 | 143.13725490196077 | 143.31210191082803 | 141.89189189189187 | 140.14084507042253 | 143.53741496598641 | 137.24137931034483 | 137.14285714285714 | 141.84397163120568 |
| 5 | 152.94117647058823 | 154.14012738853503 | 153.3783783783784 | 148.59154929577468 | 151.02040816326533 | 148.27586206896552 | 145.7142857142857 | 152.48226950354612 |
| 6 | 163.3986928104575 | 163.69426751592357 | 163.51351351351352 | 154.92957746478874 | 161.9047619047619 | 159.31034482758622 | 156.42857142857142 | 163.82978723404256 |
| 7 | 169.28104575163397 | 171.97452229299364 | 179.05405405405403 | 154.22535211267606 | 149.65986394557825 | 164.13793103448276 | 164.28571428571428 | 168.79432624113474 |
| 8 | 176.47058823529412 | 170.70063694267517 | 181.08108108108107 | 157.04225352112678 | 148.97959183673467 | 175.17241379310343 | 171.42857142857142 | 174.468085106383 |
| 9 | 186.27450980392155 | 172.61146496815286 | 177.7027027027027 | 161.9718309859155 | 148.97959183673467 | 186.20689655172413 | 175.0 | 173.75886524822695 |
| 10 | 189.5424836601307 | 180.89171974522293 | 177.02702702702703 | 167.60563380281693 | 148.97959183673467 | 186.89655172413794 | 177.85714285714283 | 179.43262411347519 |
| 11 | 186.9281045751634 | 187.2611464968153 | 182.43243243243242 | 172.53521126760566 | 156.4625850340136 | 182.75862068965517 | 182.14285714285714 | 187.94326241134752 |
| 12 | 192.81045751633985 | 193.63057324840764 | 187.16216216216216 | 178.16901408450704 | 161.9047619047619 | 182.75862068965517 | 190.7142857142857 | 191.48936170212767 |
| 13 | 197.38562091503266 | 192.35668789808918 | 185.1351351351351 | 183.80281690140848 | 172.10884353741497 | 193.10344827586206 | 197.85714285714283 | 190.0709219858156 |
| 14 | 197.38562091503266 | 194.90445859872614 | 185.8108108108108 | 184.50704225352112 | 184.35374149659867 | 193.10344827586206 | 197.14285714285717 | 185.10638297872342 |
| 15 | 193.4640522875817 | 201.91082802547774 | 188.5135135135135 | 181.69014084507046 | 185.71428571428572 | 188.9655172413793 | 194.28571428571428 | 191.48936170212767 |
| 18 | 203.26797385620915 | 210.82802547770703 | 192.56756756756758 | 191.5492957746479 | 194.55782312925172 | 195.44827586206895 | 203.57142857142856 | 192.1985815602837 |
### Chart: Muar
| Category | 1 | 2 | 3 | 4 | 5 | 6 | 7 | 8 |
|---|---|---|---|---|---|---|---|---|
| 0 | 100.0 | 100.0 | 100.0 | 100.0 | 100.0 | 100.0 | 100.0 | 100.0 |
| 1 | 113.51351351351352 | 111.46496815286623 | 114.7887323943662 | 111.11111111111111 | 110.34482758620689 | 111.9718309859155 | 114.86486486486487 | 113.1578947368421 |
| 2 | 125.0 | 121.65605095541403 | 126.7605633802817 | 122.91666666666666 | 122.75862068965517 | 124.64788732394368 | 120.94594594594592 | 124.3421052631579 |
| 3 | 133.1081081081081 | 129.93630573248407 | 134.50704225352115 | 131.24999999999997 | 133.10344827586206 | 135.2112676056338 | 133.78378378378378 | 130.26315789473685 |
| 4 | 145.94594594594594 | 142.03821656050957 | 147.88732394366198 | 138.19444444444443 | 143.44827586206895 | 142.95774647887325 | 144.59459459459458 | 140.13157894736844 |
| 5 | 152.7027027027027 | 147.13375796178346 | 152.8169014084507 | 150.0 | 152.41379310344828 | 152.8169014084507 | 153.3783783783784 | 143.42105263157896 |
| 6 | 146.6216216216216 | 157.3248407643312 | 139.43661971830988 | 154.86111111111111 | 155.17241379310346 | 160.56338028169014 | 150.67567567567568 | 126.97368421052633 |
| 7 | 123.64864864864865 | 130.57324840764332 | None | 138.88888888888889 | 140.0 | 139.43661971830988 | 123.64864864864865 | None |
| 8 | None | None | None | None | None | None | None | None |
| 9 | None | None | None | None | None | None | None | None |
| 10 | None | None | None | None | None | None | None | None |
| 11 | None | None | None | None | None | None | None | None |
| 12 | None | None | None | None | None | None | None | None |
| 13 | None | None | None | None | None | None | None | None |
| 14 | None | None | None | None | None | None | None | None |
| 15 | None | None | None | None | None | None | None | None |
| 18 | None | None | None | None | None | None | None | None |
### Chart: NS1-3Muar
| Category | 1 | 2 | 3 | 4 | 5 | 6 | 7 | 8 |
|---|---|---|---|---|---|---|---|---|
| 0 | 100.0 | 100.0 | 100.0 | 100.0 | 100.0 | 100.0 | 100.0 | 100.0 |
| 1 | 114.56953642384107 | 111.48648648648648 | 112.24489795918369 | 109.79020979020977 | 108.1081081081081 | 112.66666666666664 | 109.80392156862746 | 116.32653061224491 |
| 2 | 125.82781456953643 | 119.59459459459458 | 123.80952380952381 | 120.97902097902097 | 120.94594594594592 | 123.33333333333334 | 116.33986928104576 | 121.08843537414967 |
| 3 | 137.74834437086093 | 129.7297297297297 | 134.01360544217687 | 131.46853146853147 | 130.4054054054054 | 131.33333333333331 | 126.1437908496732 | 131.97278911564624 |
| 4 | 150.33112582781456 | 138.51351351351352 | 140.13605442176873 | 141.95804195804195 | 141.2162162162162 | 142.66666666666666 | 135.94771241830065 | 142.17687074829934 |
| 5 | 161.58940397350995 | 145.27027027027026 | 152.38095238095238 | 154.54545454545453 | 152.7027027027027 | 152.0 | 145.09803921568627 | 148.97959183673467 |
| 6 | 170.86092715231788 | 149.32432432432432 | 159.8639455782313 | 163.63636363636363 | 158.1081081081081 | 159.33333333333331 | 142.48366013071896 | 157.14285714285717 |
| 7 | 181.4569536423841 | 155.4054054054054 | 165.3061224489796 | 172.02797202797203 | 156.08108108108107 | 168.0 | 125.49019607843137 | 164.62585034013605 |
| 8 | 186.75496688741723 | 160.8108108108108 | 163.26530612244898 | 177.6223776223776 | 147.2972972972973 | 172.0 | 111.76470588235294 | 166.66666666666669 |
| 9 | 196.0264900662252 | 168.9189189189189 | 165.98639455782313 | 185.3146853146853 | 133.78378378378378 | 178.66666666666666 | None | 170.7482993197279 |
| 10 | 196.6887417218543 | 171.6216216216216 | 166.66666666666669 | 195.1048951048951 | 120.27027027027026 | 181.33333333333331 | None | 172.10884353741497 |
| 11 | 198.67549668874173 | 168.9189189189189 | 176.87074829931976 | 206.2937062937063 | None | 186.66666666666666 | None | 173.46938775510205 |
| 12 | 201.3245033112583 | 168.9189189189189 | 180.95238095238096 | 200.69930069930066 | None | 195.33333333333334 | None | 174.8299319727891 |
| 13 | 209.27152317880794 | 172.2972972972973 | 189.1156462585034 | 200.0 | None | 193.33333333333334 | None | 182.31292517006804 |
| 14 | 207.94701986754967 | 177.7027027027027 | 191.83673469387756 | 205.59440559440557 | None | 188.66666666666669 | None | 189.79591836734693 |
| 15 | 206.62251655629137 | 177.02702702702703 | 187.75510204081633 | 212.5874125874126 | None | 194.66666666666666 | None | 186.39455782312925 |
| 18 | 217.2185430463576 | 183.78378378378378 | 206.12244897959187 | 212.5874125874126 | None | 201.33333333333331 | None | 198.63945578231292 |
### Chart: 5NCMEMuar
| Category | 1 | 2 | 3 | 4 | 5 | 6 | 7 | 8 |
|---|---|---|---|---|---|---|---|---|
| 0 | 100.0 | 100.0 | 100.0 | 100.0 | 100.0 | 100.0 | 100.0 | 100.0 |
| 1 | 108.7248322147651 | 112.7659574468085 | 110.88435374149661 | 111.48648648648648 | 111.03448275862068 | 116.31205673758865 | 110.56338028169014 | 114.59854014598541 |
| 2 | 119.46308724832215 | 121.27659574468086 | 122.44897959183673 | 124.3243243243243 | 120.6896551724138 | 123.40425531914893 | 121.83098591549297 | 123.35766423357664 |
| 3 | 125.50335570469797 | 130.4964539007092 | 125.85034013605443 | 130.4054054054054 | 125.51724137931033 | 134.04255319148933 | 130.2816901408451 | 134.3065693430657 |
| 4 | 135.5704697986577 | 139.7163120567376 | 136.73469387755105 | 141.2162162162162 | 135.86206896551724 | 144.68085106382978 | 140.84507042253523 | 145.25547445255472 |
| 5 | 143.6241610738255 | 149.645390070922 | 144.89795918367346 | 145.94594594594594 | 144.13793103448276 | 151.06382978723406 | 152.8169014084507 | 152.55474452554745 |
| 6 | 136.24161073825502 | 143.26241134751774 | 153.74149659863946 | 155.4054054054054 | 133.79310344827587 | 150.354609929078 | 151.40845070422534 | 160.5839416058394 |
| 7 | None | None | 133.33333333333334 | 142.56756756756758 | 117.93103448275863 | 129.7872340425532 | 130.98591549295776 | 143.06569343065695 |
| 8 | None | None | None | None | None | None | None | None |
| 9 | None | None | None | None | None | None | None | None |
| 10 | None | None | None | None | None | None | None | None |
| 11 | None | None | None | None | None | None | None | None |
| 12 | None | None | None | None | None | None | None | None |
| 13 | None | None | None | None | None | None | None | None |
| 14 | None | None | None | None | None | None | None | None |
| 15 | None | None | None | None | None | None | None | None |
| 18 | None | None | None | None | None | None | None | None |Relative body weight (Day 0=100%)
### Chart: NS5-3NMuar
| Category | 1 | 2 | 3 | 4 | 5 | 6 | 7 | 8 |
|---|---|---|---|---|---|---|---|---|
| 0 | 100.0 | 100.0 | 100.0 | 100.0 | 100.0 | 100.0 | 100.0 | 100.0 |
| 1 | 112.67605633802818 | 112.57861635220125 | 113.57142857142857 | 111.03896103896105 | 116.78832116788323 | 112.75167785234899 | 111.11111111111111 | 110.00000000000001 |
| 2 | 124.64788732394368 | 123.89937106918238 | 122.85714285714285 | 122.07792207792207 | 128.46715328467155 | 125.50335570469797 | 120.13888888888889 | 122.0 |
| 3 | 135.2112676056338 | 137.73584905660377 | 136.42857142857144 | 131.8181818181818 | 140.14598540145985 | 136.24161073825502 | 128.4722222222222 | 130.0 |
| 4 | 145.07042253521126 | 144.0251572327044 | 148.57142857142858 | 139.6103896103896 | 150.36496350364965 | 144.96644295302013 | 140.97222222222223 | 140.0 |
| 5 | 158.45070422535213 | 150.94339622641508 | 155.71428571428572 | 146.1038961038961 | 157.66423357664237 | 153.0201342281879 | 150.69444444444443 | 146.66666666666666 |
| 6 | 170.4225352112676 | 164.1509433962264 | 165.7142857142857 | 155.84415584415586 | 164.96350364963504 | 163.75838926174495 | 164.58333333333331 | 159.33333333333331 |
| 7 | 173.943661971831 | 180.50314465408803 | 148.57142857142858 | 162.987012987013 | 155.47445255474454 | 169.7986577181208 | 162.49999999999997 | 161.33333333333331 |
| 8 | 162.6760563380282 | 178.61635220125785 | 127.14285714285715 | 168.8311688311688 | None | 177.8523489932886 | 156.94444444444443 | 166.0 |
| 9 | 151.40845070422534 | 180.50314465408803 | None | 172.07792207792207 | None | 188.59060402684565 | 145.83333333333331 | 172.0 |
| 10 | 140.84507042253523 | 184.27672955974842 | None | 176.62337662337663 | None | 186.5771812080537 | 142.36111111111111 | 175.33333333333334 |
| 11 | 145.07042253521126 | 191.8238993710692 | None | 180.5194805194805 | None | 187.24832214765098 | 142.36111111111111 | 181.33333333333331 |
| 12 | 152.11267605633805 | 199.37106918238993 | None | 187.012987012987 | None | 193.28859060402687 | 142.36111111111111 | 182.66666666666666 |
| 13 | 157.04225352112678 | 198.11320754716982 | None | 187.012987012987 | None | 200.0 | 150.0 | 180.0 |
| 14 | 161.26760563380282 | 194.9685534591195 | None | 190.25974025974025 | None | 205.36912751677852 | 152.08333333333331 | 184.0 |
| 15 | 164.08450704225353 | 201.25786163522014 | None | 190.9090909090909 | None | 203.3557046979866 | 156.94444444444443 | 188.66666666666669 |
| 18 | 173.23943661971833 | 206.91823899371067 | None | 201.2987012987013 | None | 210.73825503355704 | 175.69444444444443 | 186.66666666666666 |
### Chart: NS4A-5Muar
| Category | 1 | 2 | 3 | 4 | 5 | 6 | 7 | 8 |
|---|---|---|---|---|---|---|---|---|
| 0 | 100.0 | 100.0 | 100.0 | 100.0 | 100.0 | 100.0 | 100.0 | 100.0 |
| 1 | 112.58278145695364 | 106.993006993007 | 114.09395973154362 | 109.03225806451613 | 112.14285714285714 | 112.7659574468085 | 107.94701986754967 | 108.97435897435899 |
| 2 | 121.8543046357616 | 123.07692307692308 | 123.48993288590601 | 113.54838709677419 | 121.42857142857142 | 122.69503546099291 | 118.54304635761588 | 117.3076923076923 |
| 3 | 132.45033112582783 | 132.86713286713285 | 137.58389261744966 | 125.80645161290323 | 133.57142857142856 | 134.75177304964538 | 128.47682119205297 | 127.56410256410255 |
| 4 | 142.3841059602649 | 142.65734265734264 | 146.30872483221478 | 137.4193548387097 | 142.14285714285714 | 145.39007092198582 | 135.76158940397352 | 132.05128205128207 |
| 5 | 155.62913907284766 | 149.65034965034963 | 156.3758389261745 | 150.32258064516128 | 155.71428571428572 | 158.86524822695034 | 146.35761589403975 | 142.94871794871796 |
| 6 | 160.26490066225165 | 153.84615384615384 | 167.78523489932886 | 156.7741935483871 | 165.7142857142857 | 172.34042553191492 | 154.96688741721854 | 148.71794871794873 |
| 7 | 168.21192052980132 | 163.63636363636363 | 175.16778523489933 | 165.16129032258064 | 172.85714285714283 | 182.97872340425533 | 162.91390728476821 | 156.4102564102564 |
| 8 | 172.18543046357618 | 169.93006993006992 | 183.2214765100671 | 168.38709677419357 | 182.14285714285714 | 195.74468085106383 | 172.18543046357618 | 162.17948717948718 |
| 9 | 178.1456953642384 | 177.6223776223776 | 193.28859060402687 | 174.83870967741936 | 192.85714285714286 | 208.51063829787236 | 174.83443708609272 | 166.66666666666669 |
| 10 | 185.43046357615896 | 186.71328671328672 | 195.3020134228188 | 183.2258064516129 | 204.2857142857143 | 206.38297872340425 | 182.11920529801324 | 169.87179487179486 |
| 11 | 184.76821192052978 | 188.8111888111888 | 196.6442953020134 | 183.2258064516129 | 207.85714285714286 | 203.54609929078015 | 186.75496688741723 | 175.64102564102564 |
| 12 | 187.41721854304637 | 182.51748251748253 | 198.65771812080538 | 184.51612903225808 | 207.85714285714286 | 208.51063829787236 | 186.0927152317881 | 173.0769230769231 |
| 13 | 190.0662251655629 | 182.51748251748253 | 202.01342281879192 | 185.16129032258064 | 207.14285714285717 | 214.1843971631206 | 180.13245033112582 | 171.7948717948718 |
| 14 | 198.67549668874173 | 184.6153846153846 | 208.05369127516778 | 189.03225806451613 | 211.42857142857144 | 219.85815602836877 | 184.76821192052978 | 176.92307692307693 |
| 15 | 196.6887417218543 | 188.8111888111888 | 210.06711409395976 | 198.06451612903226 | 219.28571428571425 | 218.43971631205673 | 188.74172185430464 | 180.76923076923077 |
| 18 | 199.33774834437088 | 195.80419580419579 | 214.76510067114094 | 196.7741935483871 | 217.14285714285714 | 227.6595744680851 | 192.05298013245033 | 182.69230769230768 |Day post inoculation
Fig. S2. Body weight of mice inoculated with Mie/41/2002, Muar, or the 4 intertypic mutant viruses (experiment 1 in Table 1).

## Slide 3
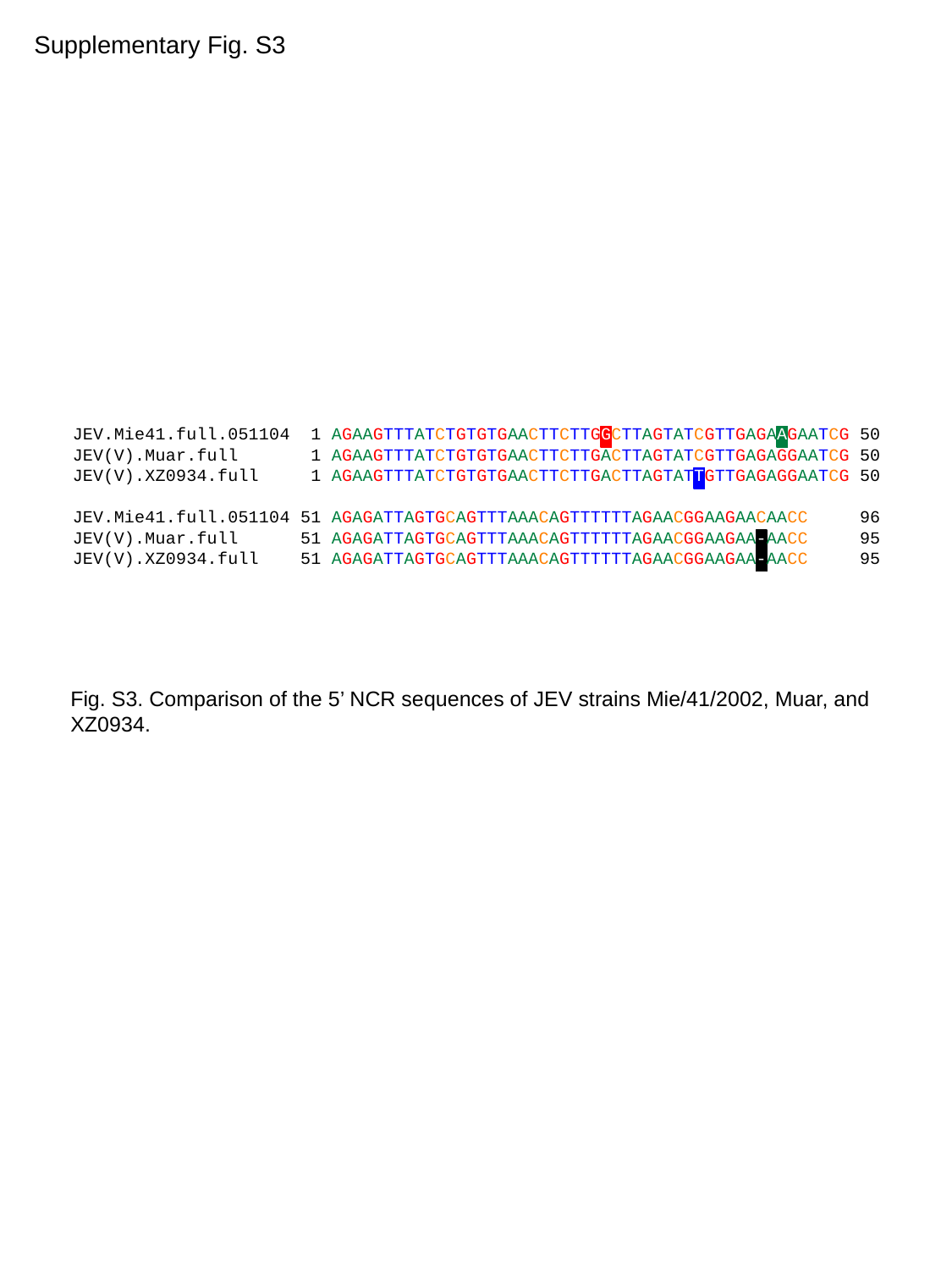

Supplementary Fig. S3
Fig. S3. Comparison of the 5’ NCR sequences of JEV strains Mie/41/2002, Muar, and XZ0934.
